# Upregulation of *NETO2* gene in colorectal cancer

**DOI:** 10.1186/s12863-017-0581-8

**Published:** 2017-12-28

**Authors:** Maria S. Fedorova, Anastasiya V. Snezhkina, Elena A. Pudova, Ivan S. Abramov, Anastasiya V. Lipatova, Sergey L. Kharitonov, Asiya F. Sadritdinova, Kirill M. Nyushko, Kseniya M. Klimina, Mikhail M. Belyakov, Elena N. Slavnova, Nataliya V. Melnikova, Maria A. Chernichenko, Dmitry V. Sidorov, Marina V. Kiseleva, Andrey D. Kaprin, Boris Y. Alekseev, Alexey A. Dmitriev, Anna V. Kudryavtseva

**Affiliations:** 1 Engelhardt Institute of Molecular Biology, Russian Academy of Sciences, Moscow, Russia; 2 National Medical Research Radiological Center, Ministry of Health of the Russian Federation, Moscow, Russia; 3 Vavilov Institute of General Genetics, Russian Academy of Sciences, Moscow, Russia

**Keywords:** Colorectal cancer, NETO2, Epithelial-mesenchymal transition, Gene expression, QPCR

## Abstract

**Background:**

Neuropilin and tolloid-like 2 (NETO2) is a single-pass transmembrane protein that has been shown primarily implicated in neuron-specific processes. Upregulation of *NETO2* gene was also detected in several cancer types. In colorectal cancer (CRC), it was associated with tumor progression, invasion, and metastasis, and seems to be involved in epithelial-mesenchymal transition (EMT). However, the mechanism of NETO2 action is still poorly understood.

**Results:**

We have revealed significant increase in the expression of *NETO2* gene and deregulation of eight EMT-related genes in CRC. Four of them were upregulated (*TWIST1, SNAIL1, LEF1*, and *FOXA2*); the mRNA levels of other genes (*FOXA1*, *BMP2*, *BMP5*, and *SMAD7*) were decreased. Expression of *NETO2* gene was weakly correlated with that of genes involved in the EMT process.

**Conclusions:**

We found considerable *NETO2* upregulation, but no significant correlation between the expression of *NETO2* and EMT-related genes in CRC. Thus, NETO2 may be involved in CRC progression, but is not directly associated with EMT.

## Background

Colorectal cancer (CRC) is the third most common malignancy in developed countries, and furthermore, its incidence rate has continuously increased over the past few decades [1]. While early-stage CRC can be effectively treated with radical surgery, approximately 20% of CRC patients present with advanced-stage disease at the time of initial diagnosis. These patients frequently have  metastases  that result in increased risk of death even after radical surgery [2]. CRC is characterized by multiple genetic and epigenetic changes that affect metabolic and signaling pathways [3–6]. For instance, cancer cells have a higher glycolytic rate than normal ones [7–9], and, as a consequence, the terminal glycolytic metabolite lactate is exported to the extracellular matrix contributing the extracellular acidosis [10]. The acidic extracellular pH (pH_*e*_), in turn, can induce epithelial-mesenchymal transition (EMT) in carcinoma models and is closely associated with tumor metastasis [11, 12]. Thus, in addition to improving the current understanding of the mechanisms underlying CRC metastasis, it is important to identify novel components of EMT process that may be the potential biomarkers of the disease progression and can further contribute to both the selection of optimal treatment options and effective treatment monitoring for patients with CRC.

*NETO2* gene is localized on chromosome 16 and encodes a transmembrane glycoprotein of unknown function. It has been shown that the abundant expression of NETO2 protein in neurons is essential for proper neurological function [13, 14]. Initially, NETO2 was believed to be a brain-specific protein [15, 16]; however, recent studies described overexpression of *NETO2* in several types of cancer, including renal, lung, colon, and cervical carcinomas [17]. Accordingly, Hu et al. recently suggested high expression of *NETO2* as a potential biomarker of both advanced tumor progression and poor prognosis in patients with CRC [18].

In the present study, we hypothesized that the association of *NETO2* overexpression with tumor progression, invasion, and metastasis may be indicative of its involvement in the epithelial-mesenchymal transition in CRC. To investigate the validity of this hypothesis, we evaluated whether *NETO2* expression was correlated with that of genes established to mediate the EMT process.

## Methods

### Tissue samples

A total of 44 CRC and matched morphologically normal tissue samples, which were obtained after surgical resection, but prior to patient treatment with radiation and/or chemotherapy, were frozen and stored in liquid nitrogen until use. All CRC samples were classified according to the American Joint Committee on Cancer (AJCC) staging system [19], and only those samples comprising 70% or more tumor cells were selected for analysis. Written informed consent was obtained from all patients for participation in the present study, which was approved by Herzen Moscow Cancer Research Institute - branch of National Medical Research Radiological Center, Ministry of Health of Russia Federation (Moscow, Russia), and conducted in strict accordance with the principles outlined in the Declaration of Helsinki (1964). Clinicopathologic characteristics of the CRC patients are shown in Table 1.

**Table 1:** Clinicopathologic characteristics of the CRC patients

Characteristic	Total, n
Gender	
Male	23
Female	21
Age (years)	
≤ 60	14
> 60	30
Clinical stage	
I	2
II	11
III	15
IV	16
Distant metastases (Stage IV)	
Negative	4
Positive	12

### RNA isolation and cDNA synthesis

Total RNA was isolated from the frozen tissue samples using RNeasy Mini kit (Qiagen, Germany) according to manufacturer’s instructions. RNA quality was measured *via* the RNA Integrity Number (RIN) method using an Agilent RNA Bioanalyzer 2100 (Agilent Technologies, USA). RNA quantification was performed using a NanoDrop 1000 instrument (NanoDrop Technologies, USA). cDNA was synthesized from the isolated RNA using M-MLV Reverse Transcriptase (Thermo Fisher Scientific, USA) and random hexamers.

### Quantitative PCR (qPCR)

Quantitative polymerase chain reaction was performed using TaqMan Assay (Thermo Fisher Scientific) primers and probes for target genes (*NETO2*: Hs00983152_m1, *TWIST1*: Hs00361186_m1, *SNAIL1*: Hs00195591_m1, *SNAIL2*: Hs00161904_m1, *ZEB1*: Hs01566408_m1, *ZEB2*: Hs00207691_m1, *LEF1*: Hs01547250_m1, *FOXA2*: Hs00232764_m1, *FOXA1*: Hs04187555_m1, *CDH1*: Hs01023895_m1, *STAT1*: Hs00374280_m1, *BMP2*: Hs00154192_m1, *BMP5*: Hs00234930_m1, *VIM*: Hs00958111_m1, *SMAD2*: Hs00998187_m1, *SMAD3*: Hs00969210_m1, *SMAD4*: Hs00929647_m1, *SMAD7*: Hs00998193_m1). Primers and probes for reference genes, *GUSB* and *RPN1*, were previously described [20, 21]. All qPCRs were carried out in triplicate in total reaction volume of 20 μL using an AB 7500 Real-Time PCR System (Thermo Fisher Scientific) to achieve cycling conditions comprising 95 °C for 10 min, followed by 40 cycles of 95 °C for 15 s, 60 °C for 60 s, and 72 °C for 30 s.

QPCR data were analyzed using Relative Quantitation (Thermo Fisher Scientific) software and ATG program taking into account the efficiency of the PCR amplification [22, 23]. The expression levels of target genes were normalized to those of the reference genes. Finally, relative (T/N) expression level of target genes was calculated using the ΔΔCt method [24]. Since the relative inner variability between the calculated mRNA levels of the reference genes was found to be less than two-fold, a variation in the expression of the target genes of two-fold or greater was considered to be significant.

### Statistical analysis

Inter- and intra-group comparisons were performed using non-parametric Wilcoxon/Mann-Whitney and Kruskal-Wallis tests. Spearman’s rank correlation coefficient (*r*_*s*_) was used for revealing correlations between *NETO2* and EMT-related gene expression. All statistical analyses were performed using PASW Statistics 18 (SPSS Inc., USA) software. A *p*-value < 0.05 was considered to indicate statistical significance.

## Results

### Upregulation of *NETO2* gene in CRC

**Fig. 1:**
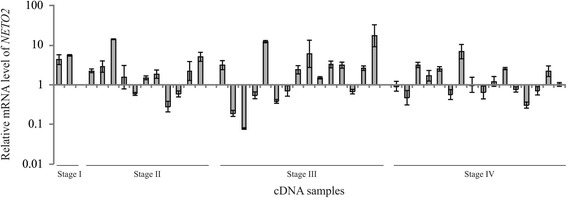
Relative mRNA level of *NETO2* gene in CRC. QPCR data

QPCR analysis of the relative *NETO2* mRNA level across the 44 CRC samples revealed that *NETO2* expression was increased by a factor of 2–50 in 41% (18/44) of cases (Fig. 1). In contrast, *NETO2* expression was decreased by a factor of 2–25 in 14% (6/44) of CRC samples. These results demonstrating a significant increase in the expression of *NETO2* in the analyzed CRC samples are consistent with those of the previous study by Oparina et al. [17].

### Deregulation of EMT-related genes in CRC

We performed an analysis of the relevant literature and selected 17 genes related to EMT process in CRC (Table 2). Using qPCR, mRNA levels of these genes were analyzed in 44 CRC samples (Table 3).

**Table 2:** Genes involved in the epithelial-mesenchymal transition in CRC

Gene	Description	References
*TWIST1*	TWIST1 is a highly conserved basic helix-loop-helix (bHLH) transcription factor that regulates the EMT required for neural crest migration during vertebrate embryonic development. *TWIST1* expression is positively associated with patient survival after curative CRC resection, and thus is a promising candidate biomarker of the disease progression.	[33] [34] [35]
*SNAIL1*	SNAIL1 is a transcriptional regulator of E-cadherin, which suppression is critical to facilitate the EMT process. *SNAIL1* mRNA level is not detectable in the normal colon mucosa, but is upregulated in 60–70% of CRC. Importantly, aberrant *SNAIL1* expression in CRC has been shown to be associated not only with poor patient prognosis, but also with a reduced relapse-free survival time.	[36] [37] [38] [39] [40]
*SNAIL2*	SNAIL2 has been implicated as an anti-apoptotic factor, and is thought to mediate the EMT process by repressing E-cadherin transcription. Accordingly, *SNAIL2* expression in human CRC cell lines has been shown to be correlated with critical EMT properties, including the loss of E-cadherin expression and an increase in both cell migration and invasion.	[41] [42]
*ZEB1*	ZEB1 mediates the EMT pathway, and in fact has been shown to be not only sufficient to induce the EMT, but also necessary for maintaining the adapted mesenchymal phenotype. ZEB1 contains zinc finger clusters in both its N- and C-terminal regions, and a homeodomain in the central region. In CRC cells, ZEB1 has been shown to critically mediate the EMT, and thus may be an important regulator of CRC metastasis.	[43] [44] [45]
*ZEB2*	ZEB2 is a member of the Zfh1 family of two-handed zinc-finger transcription factors. It is frequently expressed in colon cancer, and has been shown by several previous studies to induce the EMT, and to facilitate cancer-cell metastasis, possibly *via* the repression and upregulation of E-cadherin and vimentin respectively.	[46] [47] [48]
*LEF1*	LEF1 is critical for tumor-cell adhesion and/or migration, and thus, also for tumor invasion and metastasis. In addition, it plays a pivotal role in carcinogenesis and CRC progression, partly *via* its function in the LEF1/β-catenin complex, which is a crucial effector of the Wnt signaling pathway. Increased *LEF1* expression has been shown to be correlated with node and distant metastasis, and with an advanced tumor stage. Furthermore, *LEF1* was shown to be involved in CRC invasion and metastasis.	[49] [50] [51]
*FOXA1* and *FOXA2*	Forkhead box (FOX) protein A1 (FOXA1) is a transcription factor belonging to the FOX gene superfamily that mediates fundamental developmental and differentiation processes. Specifically, it modulates transcriptional programs in a tissue-dependent manner by inducing nucleosomal rearrangement, and by altering chromatin accessibility to the transcriptional machinery. *FOXA1* has been shown to be overexpressed in CRC, and furthermore, to be positively associated with poor clinicopathological features. This suggests that its expression may be a promising candidate prognostic biomarker for patients with CRC. *FOXA2* is a known key regulator of CRC metastasis to the liver.	[52] [53] [54] [55]
*CDH1*	*CDH1* gene encodes a classical cadherin. The E-cadherin-mediated cell adhesion system is required for both the EMT, and for cellular invasion, angiogenesis, and metastatic/tumor progression in many cancers, including CRC.	[56]
*STAT1*	STAT1 is a signal mediator that controls cell-death functions in the context of both pro-apoptotic and anti-proliferative interferon-dependent signaling. It appears to exhibit tumor suppressive functions, and its activity has been shown to be associated with a favorable patient prognosis in some cancers.	[57] [58]
*BMP2* and *BMP5*	Bone morphogenetic proteins (BMPs) are the secreted ligands of the proteins belonging to the transforming growth factor beta superfamily (TGFβ), and are important regulators of body-axis patterning during embryogenesis. In adult tissues, they regulate cell growth, apoptosis, and differentiation. The biological effects of BMPs have been predominantly studied in mesoderm-derived cells and tissues, and to a lesser degree, in epithelial cells and tissues. In general, BMPs are involved in the regulation of cancer progression and metastasis possibly through TGF-β-induced SMAD3-dependent EMT. Inactivation of BMP signaling increases the tumorigenicity of normal colon stem cells.	[59] [60] [61]
*VIM*	*VIM* is a Wnt-targeted gene that is expressed in normal mesenchymal cells, and that encodes the intermediate filament protein, vimentin. Previous studies have shown that vimentin mediates both cellular structure and integrity. Furthermore, vimentin has also been demonstrated to mediate cell shape and motility during the EMT process, which is required for cancer-cell metastasis.	[62] [63] [64] [65]
*SMAD2, SMAD3, SMAD4,* and *SMAD7*	The SMADs are a family of structurally related signaling proteins that can be divided into three subgroups according to their respective functions in TGFβ signaling. Specifically, the receptor-activated SMADs, including SMAD2 and SMAD3, are serine-phosphorylated following TGF-receptor complex formation. The unique SMAD4 co-SMAD (which is common to both TGFβ and BMP signaling), then interacts with the phosphorylated SMAD2/SMAD3. The resulting heteropolymer migrates to the nucleus and complexes with tissue-specific transcription factors, thereby inducing the transcription of TGFβ target genes, including *SMAD7*. Finally, *SMAD7*, which is the only TGFβ-specific anti-SMAD, prevents SMAD2/3 activation, thereby providing a transient TGFβ response in the form of a negative feedback loop. Immunohistochemical analysis has revealed the expression of SMADs during EMT process in CRC.	[66] [67] [68] [69]

**Table 3:** Relative mRNA levels of EMT-related genes in CRC

Gene	Frequency of mRNA level changes, %		Median of mRNA level changes, n-fold
	↑ increased expression	↓ decreased expression	
*TWIST1*	**68** (30/44)	5 (2/44)	2.8↑
*SNAIL1*	**80** (35/44)	2 (1/44)	3.3↑
*SNAIL2*	11 (5/44)	20 (9/44)	1.2↓
*ZEB1*	9 (4/44)	**36** (16/44)	1.5↓
*ZEB2*	7 (3/44)	**45** (20/44)	1.7↓
*LEF1*	**75** (33/44)	2 (1/44)	3.9↑
*FOXA1*	7 (3/44)	**52** (23/44)	2.1↓
*FOXA2*	**59** (26/44)	5 (2/44)	2.5↑
*CDH1*	5 (2/44)	16 (7/44)	1.3↓
*STAT1*	25 (11/44)	5 (2/44)	1.4↑
*BMP2*	2 (1/44)	**75** (33/44)	3.2↓
*BMP5*	7 (3/44)	**84** (37/44)	7.6↓
*VIM*	18 (8/44)	7 (3/44)	1.3↑
*SMAD2*	0 (0/44)	11 (5/44)	1.4↓
*SMAD3*	0 (0/44)	11 (5/44)	1.2↓
*SMAD4*	0 (0/44)	23 (10/44)	1.5↓
*SMAD7*	0 (0/44)	**43** (19/44)	1.8↓

*Note:* Significant frequencies (*p* < 0.05) are marked in bold

#### *TWIST1* gene

Up to 26-fold increase in the expression of *TWIST1* gene was revealed in the majority (68%, 30/44) of CRC samples compared to matched normal tissues. In contrast, two CRC samples exhibited decreased *TWIST1* expression from 4- to 6-fold. The mean value of relative mRNA level of *TWIST1* gene was 2.8.

#### *SNAIL1* and *SNAIL2* genes

Quantitative analysis of *SNAIL1* expression showed it to be significantly increased in 80% (35/44) of CRC cases. mRNA level of *SNAI1* gene was decreased by a factor of 6 only in one sample. The expression of *SNAIL2* was found to be decreased by a factor of 2–25 in 20% (9/44) of CRC samples, and increased by a factor of 2–3 in 11% (5/44) of ones. The mean value of relative mRNA levels of *SNAI1* and *SNAIL2* genes were 3.3 and 1.2, respectively.

#### *ZEB1* and *ZEB2* genes

Analysis of *ZEB1* gene expression revealed it to be decreased by a factor of 2–48 in 36% (16/44) and increased in 9% (4/44) of CRC samples. The expression of *ZEB2* gene was decreased in 45% (19/44) and increased in 7% (3/44) of CRC cases. The mean value of relative mRNA levels of *ZEB1* and *ZEB2* genes were 1.5 and 1.7, respectively.

#### *LEF1* gene

*LEF1* gene expression was increased by a factor of 2–52 in 75% (33/44) of CRC cases, and slightly decreased by a factor of two only in one sample. The mean value of relative mRNA level of *LEF1* gene was 3.9.

#### *FOXA1* and *FOXA2* genes

The analysis of *FOXA1* and *FOXA2* gene expression showed that while *FOXA1* expression was decreased by a factor of 2–79 in 52% (23/44) of CRC samples, *FOXA2* expression was increased from 2- to 23-fold in 59% (26/44) of cases. Up to 4-fold increase in the expression of *FOXA1* gene was detected in 7% (3/44) of examined samples. *FOXA2* gene was downregulated by a factor of 2–70 in 5% (2/44) of CRC cases. The mean value of relative mRNA levels of *FOXA1* and *FOXA2* genes were 2.1 and 2.5, respectively.

#### *CDH1* gene

The analysis of *CDH1* gene expression showed it to be decreased by a factor of 2–86 in 16% (7/44) of CRC samples, and increased by a factor of two in 5% (2/44) of cases. The mean value of relative mRNA level of *CDH1* gene was 1.3.

#### *STAT1* gene

Quantification of *STAT1* gene expression revealed it to be increased by a factor of 2–4 in 25% (11/44) of cases, and decreased by a factor of 3 in 5% (2/44) of CRC samples. The mean value of relative mRNA level of *STAT1* gene was 1.4.

#### *BMP2* and *BMP5* genes

The expression of both *BMP2* and *BMP5* genes was suppressed in 75% (33/44) and 84% (37/44) of examined CRC samples, respectively. Increase in the *BMP2* gene expression was shown in only one sample (2%), while that of *BMP5* gene was detected in 7% (3/44) of CRC cases. The mean value of relative mRNA levels of *BMP2* and *BMP5* genes were 3.2 and 7.6, respectively.

#### *VIM* gene

The analysis of *VIM* expression showed it to be increased by a factor of 2–6 in 18% (8/44) of CRC samples, and decreased by a factor of 2–4 in 7% (3/44) of cases. The mean value of relative mRNA level of *VIM* gene was 1.3.

#### *SMAD2, SMAD3, SMAD4,* and *SMAD7* genes

QPCR analysis showed *SMAD2, SMAD3, SMAD4,* and *SMAD7* mRNA levels to be decreased by a factor of 2–10 in 11–43% of the examined CRC samples. The mean value of relative mRNA levels of *SMAD2, SMAD3, SMAD4,* and *SMAD7* genes were 1.4, 1.2, 1.5, and 1.8, respectively.

### mRNA level of *NETO2* is not correlated with that of EMT-related genes in CRC

We used the Spearman’s correlation coefficient to test the proposed hypothesis that *NETO2* mRNA level in CRC correlates with that of the EMT-related genes. The results of this analysis showed that across the 44 analyzed CRC samples, 17 association pairs were identified between *NETO2* and various genes involved in EMT, all of which exhibited weak relationship (Table 4). The most significant correlations were determined between *NETO2* and *SMAD7* expression (*r*_*s*_ = 0.25, *p* < 0.05) and between *NETO2* and *TWIST1* expression (*r*_*s*_ = −0.24, *p* < 0.05). These results indicate that the expression of *NETO2* in CRC is only weakly correlated with that of EMT-related genes.

**Table 4:** Spearman’s correlation coefficients between mRNA levels of *NETO2* and EMT-related genes

Gene	Spearman’s correlation coefficient, *r*_*s*_
	*NETO2*
*TWIST1*	−0.24
*SNAIL1*	−0.12
*SNAIL2*	−0.07
*ZEB1*	−0.05
*ZEB2*	0.03
*LEF1*	0.06
*FOXA1*	0.06
*FOXA2*	0.06
*CDH1*	0.11
*STAT1*	0.12
*BMP2*	0.12
*BMP5*	0.14
*VIM*	0.18
*SMAD2*	0.21
*SMAD3*	0.23
*SMAD4*	0.24
*SMAD7*	0.25

## Discussion

The *NETO2* gene encodes a transmembrane protein that is predominantly expressed in normal brain and retinal tissues. Thus, previous studies have primarily focused on NETO2 function in the context of neurobiology; *in vitro* analyses have revealed that NETO2 interacts with the GluK2 and GluK5 subunits of kainate receptors to significantly enhance kainate receptor-mediated signaling [25]. Recently, NETO2 has been shown to be involved in carcinogenesis. In a mutant cell line overexpressing metastasis-suppressor gene *NM23-H1,* which can reduce the metastatic potential of various types of cancer cells, *NETO2* was amongst the nine genes identified to exhibit increased mRNA level [26]. *NETO2* expression was reported to be upregulated in proliferating pediatric hemangiomas [27]. Notably, we previously demonstrated that *NETO2* mRNA level is frequently overexpressed in kidney and lung cancers, and resultantly suggested it as a potential marker to early diagnosis of these diseases [17]. Hu and co-authors suggested both the potential significance of *NETO2* expression in CRC carcinogenesis and its clinical relevance to the disease progression, invasion, and metastasis [18].

The EMT process is well established to be required not only for embryonic development, but also for cancer progression and metastasis, since it facilitates the acquisition of invasive properties that allow cancer cells to enter the surrounding stroma and thereby generate a favorable tumor microenvironment [28–30]. Moreover, EMT process is known to be closely associated with cancer recurrence and chemoresistance. Nevertheless, the mechanisms underlying the involvement of EMT process in these events seem to vary significantly between cancer types.

To date, *NETO2* is known to be associated with poor prognosis and metastasis in CRC, but not with the occurrence of EMT in this context. Thus, the present study investigated whether *NETO2* expression in CRC was correlated with that of key genes involved in the EMT, including *TWIST1, SNAIL1, SNAIL2, ZEB1, ZEB2, LEF1, FOXA2, FOXA1, CDH1, STAT1, BMP2, BMP5, VIM, SMAD2, SMAD3, SMAD4,* and *SMAD7*. The results obtained in the work confirmed that *NETO2* is overexpressed in CRC. It has also been demonstrated that several genes involved in the EMT process were upregulated in CRC compared to matched normal tissues, including *TWIST1, SNAIL1, LEF1*, and *FOXA2,* which mRNA levels were increased by an average factor of 2.8, 3.3, 3.9, and 2.5 (median) respectively. Conversely, the mRNA levels of *FOXA1*, *BMP2*, *BMP5,* and *SMAD7* genes were found to be decreased by a factor of 2.1, 3.2, 7.6, and 1.8 (median) respectively that is again in concordance with the results of recently studies [31, 32].

Notably, we found no significant correlation between the expression of *NETO2* gene and that of the analyzed EMT-related genes in CRC. Thus, it is likely that *NETO2* is involved in CRC progression, but is not directly associated with EMT.

## Conclusions

*NETO2* expression was found to be considerably increased, but not significantly correlated with the mRNA levels of EMT-related genes in CRC. Thus, while *NETO2* overexpression may be indicative of poor clinical prognosis and metastasis, this is unlikely to be a direct result of alterations in the EMT process. Certainly, the molecular basis for and biological relevance of *NETO2* upregulation in CRC requires further investigation.
